# Supplementary material for: Zn2+-dependent DNAzymes that cleave all combinations of ribonucleotides
Source: Commun Biol. 2021 Feb 16;4:221. doi: 10.1038/s42003-021-01738-6 (PMC7886857; doi:10.1038/s42003-021-01738-6)
Supplement: Supplementary file 2 — Supplementary Information [file 42003_2021_1738_MOESM2_ESM.pdf]

Supplementary Information

Zn^{2+}-dependent DNAzymes that cleaves all combinations of ribonucleotides

Rika Inomata, Jing Zhao and Makoto Miyagishi

Supplementary Figure 1. The LC-MS spectrum and the products of the substrate by ZincDz1 and ZincDz2.... S2

Supplementary Figure 2. The multiple turnover cleavage by ZincDz1 and ZincDz2..S3

Supplementary Figure 3. Estimation of the number of Zn^{2+} ions bound to ZincDz1 and ZincDz2...........................S4

Supplementary Figure 4. The Pb^{2+} ion selectivity of ZincDz1 and ZincDz2...S5

Supplementary Figure 5. The monovalent metal ion selectivity of ZincDz1 and ZincDz2......................................S6

Supplementary Figure 6. The Zn^{2+} ion concentration dependency of ZincDz2 for the substrate with 5´-rCCA-3´....S7

Supplementary Table 1. The list of the reported RNA-cleaving DNAzymes...S8

Supplementary Table 2. Sequences of oligonucleotides used in this study..S9

Supplementary References S11

Supplementary Figure. 1. The LC-MS spectroscopic analysis of the cleaved substrates by ZincDz1 and ZincD2.

		LC-MS	
		Calculated (Da)	Observed (Da)
ZincDz1	5'-Fragment 1	6205.0	6204.3
	5'-Fragment 2	6222.0	6222.4
	3'-Fragment	2752.9	2753.0
ZincDz2	5'-Fragment 1	5570.6	5569.8
	5'-Fragment 2	5587.6	5587.6
	3'-Fragment	3387.3	3387.6

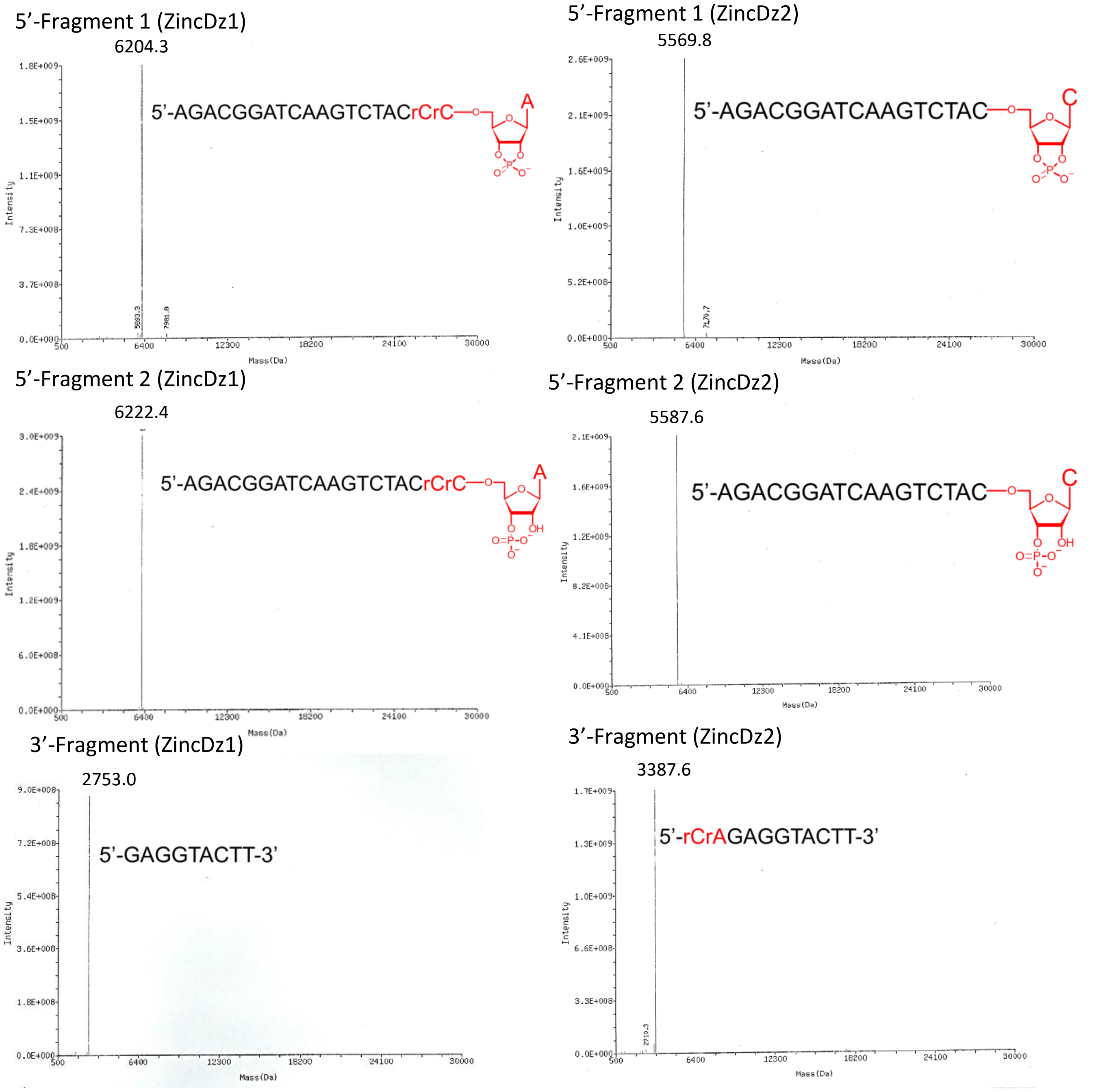

Supplementary Figure. 2. The multiple turnover cleavage by ZincDz1 and ZincDz2.

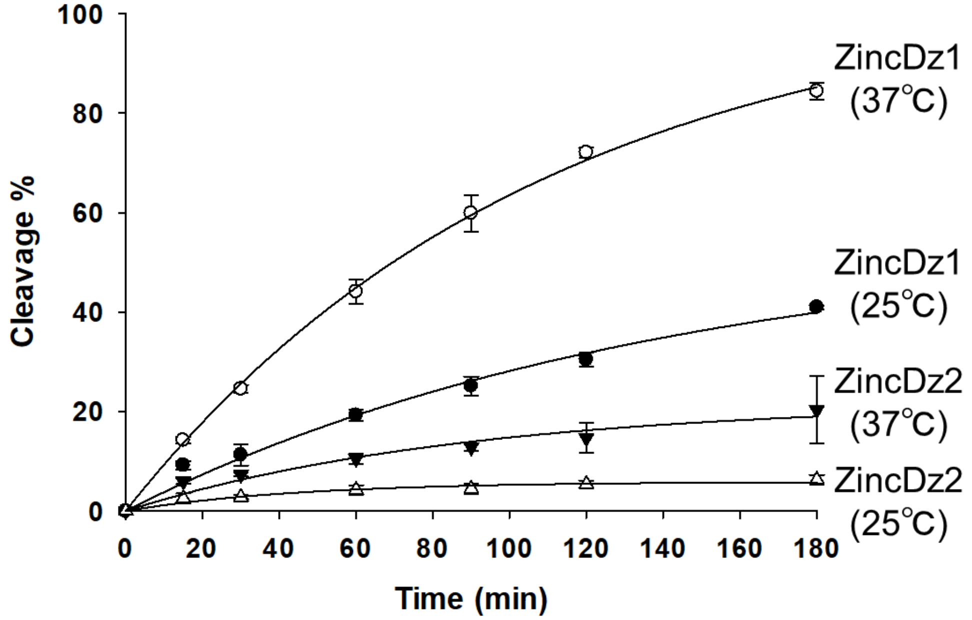

The multiple turnover cleavage reactions were performed at 25°C and 37°C with 0.1 μM ZincDz1 or ZincDz2, and 2 μM the substrate under a multiple turnover condition (20-fold excess of DNAzyme to substrate) in the reaction buffer (150 mM NaCl, 0.5 mM $ZnCl_2$ and 50 mM HEPES, pH 7.5 for ZincDz1, 150 mM NaCl, 0.8 mM $ZnCl_2$ and 50 mM HEPES, pH 7.4 for ZincDz2). The error bars represent the standard deviation (SD) from the average of three independent experiments.

Supplementary Figure. 3. Estimation of the number of Zn^{2+} ions bound to ZincDz1 and ZincDz2.

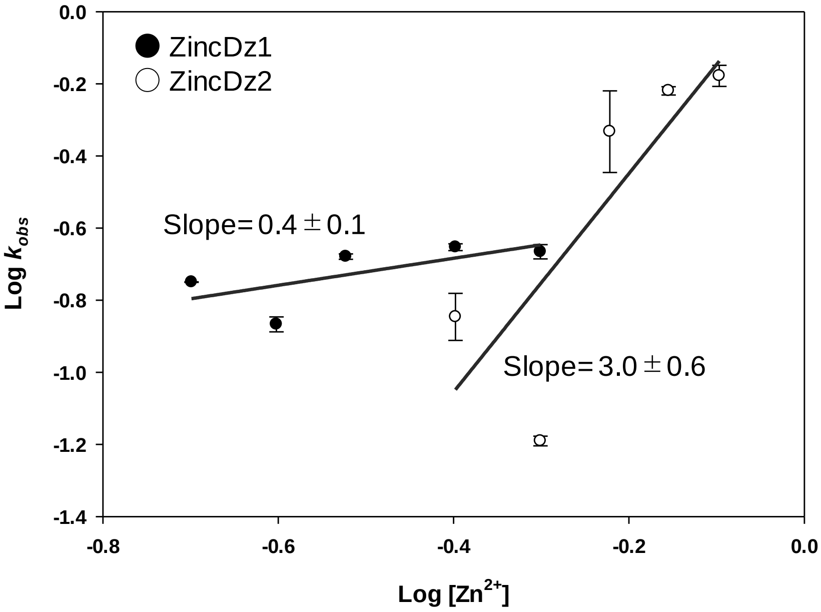

The k_{obs} of each DNAzymes was calculated from a time course experiment (1 min, 3 min, 10 min, 30 min, and 60 min) using the function described in the method section. The reaction was performed in the buffer (150 mM NaCl and 50 mM HEPES, pH 7.5 or pH 7.4) with 0.2 mM, 0.25 mM, 0.3 mM, 0.4 mM or 0.5 mM $ZnCl_2$ (for ZincDz1) or 0.4 mM, 0.5 mM, 0.6 mM, 0.7 mM or 0.8 mM $ZnCl_2$ (for ZincDz2) at 37°C. The logarithms of k_{obs} vs. logarithms of Zn^{2+} concentrations were plotted on a graph to obtain the slope of lines. The error bars represent the standard deviation (SD) from the average of three independent experiments at each point.

Supplementary Figure. 4. The Pb^{2+} ion selectivity of ZincDz1 and ZincDz2.

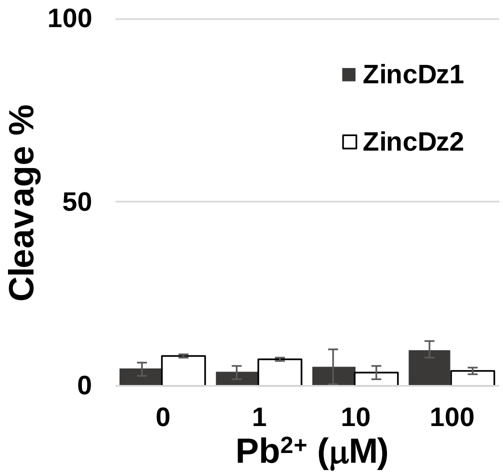

The substrate (sub-CCA) was cleaved by ZincDz1 or ZincDz2 under the single turnover condition (DNAzyme: Substrate=10: 1) in the buffer (150 mM NaCl and 50 mM HEPES, pH 7.5) with 1 μM, 10 μM or 100 μM $PbCl_2$, The error bars represent the standard deviation (SD) from the average of three independent experiments at each point.

Supplementary Figure. 5. The monovalent metal ion selectivity of ZincDz1 and ZincDz2.

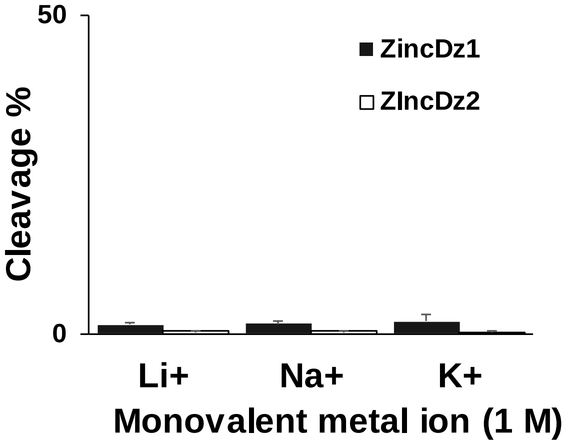

The monovalent metal ion selectivity of ZincDz1 and ZincDz2 was examined under the single turnover condition (DNAzyme: Substrate=10: 1). The substrate (sub-CCA) was cleaved by ZincDz1 or ZincDz2 in the buffer (150 mM NaCl and 50 mM HEPES, pH 7.5) with 1 M LiCl, 1 M NaCl, and 1 M KCl) at 37°C for 1 hour. The error bars represent the standard deviation (SD) from the average of three independent experiments at each point.

Supplementary Figure. 6. The Zn^{2+} ion concentration dependency of ZincDz2 for the substrate with 5´-rCCA-3´.

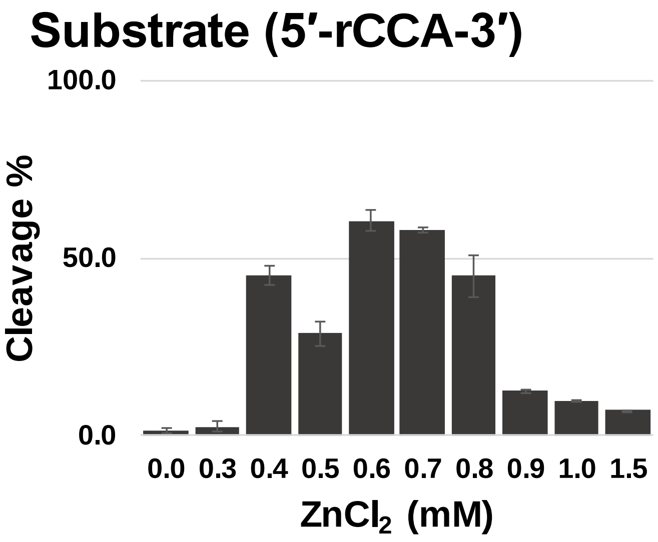

Zn^{2+} concentration dependency of ZincDz2 was examined under the single turnover condition (DNAzyme: Substrate=10: 1).The substrate with 5´-rCCA-3´ (sub-rCCA) was cleaved by ZincDz2 in the buffer (150 mM NaCl and 50 mM HEPES, pH 7.4) including 0.3 mM, 0.4 mM, 0.5 mM, 0.6 mM, 0.7 mM, 0.8 mM, 0.9 mM, 1.0 mM or 1.5 mM $ZnCl_2$ at 37°C for 1 hour. The error bars represent the standard deviation (SD) from the average of three independent experiments at each point.

Supplementary Table 1. The list of the reported RNA-cleaving DNAzymes.

DNAzyme	Core substrate (5´→3´)	Cofactor	k_{obs} (min^{-1})	Conditions
8-17[3-6]	All but rC^rU	Mg^{2+}, Pb^{2+}, Zn^{2+}	0.5~0.9	10 mM $MgCl_2$, 1 M NaCl, pH 7.5 (ref. 3)<br>400 mM KCl, 100 mM NaCl, 7.5 mM $MgCl_2$, 7.5 mM $MnCl_2$, pH 7.0 (ref. 4 and 6)<br>200 µM Pb^{2+}, 100 mM $NaNO_3$, pH 5.0 (ref.5 and 10)<br>0.1 mM $ZnCl_2$, 500 mM NaCl, pH 7.0 (ref.5)
10-23[3,6,7]	rR^rY	Mg^{2+}	0.28	10 mM $MgCl_2$, 1 M NaCl, pH 7.5 (ref. 3)<br>400 mM KCl, 100 mM NaCl, 7.5 mM $MgCl_2$, 7.5 mM $MnCl_2$, pH 7.0 (ref. 6)<br>10 mM $MgCl_2$, 50 mM NaCl, 250 mM NH_4Cl, or 2 mM spermine, pH 7.5 (ref. 7)
E6[8]	NrA^N	Mg^{2}, Pb^{2+}	0.01, 0.02	1 mM $MgCl_2$ or 1 mM $PbCl_2$, ~1 M NaCl and 50 mM HEPES, pH 7.0 (ref. 8)
GR5[9]	rA^GGA	Pb^{2+}	~0.86	0.1 µM Pb^{2+}, 25 mM NaCl, pH 7.5. (ref. 9)
17E[10]	rA^G	Pb^{2+}, Zn^{2+}	5.75, 1.35	0.1 mM $PbCl_2$ or 10 mM $MgCl_2$,100 mM $NaNO_3$, pH 5.0 (ref. 10)
17EV1[11]	rA^G	Ca^{2+}	0.52	2.5 mM $CaCl_2$, 140 mM NaCl and 50 mM Tris, pH 7.5 (ref. 11)
Bipatite II[6,12]	rA^rArNrNrN	Mg^{2+}	0.68	30 mM $MgCl_2$, 150 mM NaCl, pH 7.4 (ref. 6, 12)
PbE22[13]	rA^GGAAGA	Pb^{2+}	0.028	25 mM NaCl, pH 6.0 (ref. 13)
S4[6]	rU^rCrGrU	Mg^{2+} and Mn^{2+}	0.04	7.5 mM $MgCl_2$, and 7.5 mM $MnCl_2$, 100 mM KCl, 400 mM NaCl, pH 7.0 (ref. 6)
S9[6]	rC^rCrGrU	Mg^{2+} and Mn^{2+}	0.12	7.5 mM $MgCl_2$, and 7.5 mM $MnCl_2$, 100 mM KCl, 400 mM NaCl, pH 7.0 (ref. 6)
S15[6]	rC^TG	Mg^{2+} and Mn^{2+}	0.1	7.5 mM $MgCl_2$, and 7.5 mM $MnCl_2$, 100 mM KCl, 400 mM NaCl, pH 7.0 (ref. 6)
S21[6]	rU^rUrUrGrU	Mg^{2+} and Mn^{2+}	0.15	7.5 mM $MgCl_2$, and 7.5 mM $MnCl_2$, 100 mM KCl, 400 mM NaCl, pH 7.0 (ref. 6)
CT10.3.29M[14]	CTrX^NGGT	Mg^{2+} and Mn^{2+}	~1.4	15 mM $MgCl_2$, and 15 mM $MnCl_2$, 200 mM KCl, 800 mM NaCl, pH 7.0 (ref. 14)
Ce13d[15]	rA^GGAAG	Ce^{3+}	0.25	10 µM $CeCl_3$, 25 mM NaCl, pH 6.0 (ref. 15)
Tm7[16]	CTATrA^G	Er^{3+}, Y^{3+}	1.6	10 µM Ln^{3+} or 10 µM Y^{3+}, 25 mM NaCl, pH 6.0 (ref. 16)
NaA43[17]	TrA^GGAA	Na^{+}	0.11	400 mM NaCl, 90 mM LiCl, pH 7.0 (ref. 17)
EtNa[18]	TrA^GG	Na^{+}	0.03	120 mM NaCl, 54% Ethanol, pH 6.0 (ref. 18)
Ag10c[19]	rA^GG	Ag^{+}	0.41	10 µM $AgNO_3$,25 mM $NaNO_3$, pH 6.0 (ref. 19)

Supplementary Table 2. Sequences of oligonucleotides used in this study.

**Library used for in vitro selection experiments (5´→3´)**	
N16	ACGTTTGCTACATACCTCNNNNNNNNNNNNNNNNGTAGACTTGATCCGTCT

**Primers used for amplification (5´→3´)**	
Library-F	GTGGAGAGGTTCTTACA ACGTTTGCTACATACCTC
Library-R	GCGGAGAGGCTCTCACA AGACGGATCAAGTCTAC

**DNAzymes used in this study (5´→3´)**	
ZincDz1	AAGTACCTC GTAGGTATTAGCTAGG GTAGACTTGATC
ZincDz2	AAGTACCTC TTAGTTTATGGTTGGG GTAGACTTGATC
ZincDz2-T1A	AAGTACCTC ATAGTTTATGGTTGGG GTAGACTTGATC
ZincDz2-T2C	AAGTACCTC TCAGTTTATGGTTGGG GTAGACTTGATC
ZincDz2-A3G	AAGTACCTC TTGGTTTATGGTTGGG GTAGACTTGATC
ZincDz2-G4A	AAGTACCTC TTAATTTATGGTTGGG GTAGACTTGATC
ZincDz2-T6C	AAGTACCTC TTAGTCTATGGTTGGG GTAGACTTGATC
ZincDz2-A8T	AAGTACCTC TTAGTTTTTGGTTGGG GTAGACTTGATC
ZincDz2-T9C	AAGTACCTC TTAGTTTACGGTTGGG GTAGACTTGATC
ZincDz2-G10A	AAGTACCTC TTAGTTTATAGTTGGG GTAGACTTGATC
ZincDz2-G11A	AAGTACCTC TTAGTTTATGATTGGG GTAGACTTGATC
ZincDz2-T12C	AAGTACCTC TTAGTTTATGGCTGGG GTAGACTTGATC
ZincDz2-T13C	AAGTACCTC TTAGTTTATGGTCGGG GTAGACTTGATC
ZincDz2-G14A	AAGTACCTC TTAGTTTATGGTTAGG GTAGACTTGATC
ZincDz2-G15A	AAGTACCTC TTAGTTTATGGTTGAG GTAGACTTGATC
ZincDz2-G16A	AAGTACCTC TTAGTTTATGGTTGGA GTAGACTTGATC
ZincDz2-155CUA	AACCCCTATCACGAT TTAGTTTATGGTTGGG CATTAA
ZincDz2-155UAA	AACCCCTATCACGA TTAGTTTATGGTTGGA GCATTAA
ZincDz2-155GUG	AACCCCTAT TTAGTTTATGGTTGGC GATTAGCATTAA
ZincDz2C	AAGTACCTC TTAGTTTATGGTTGGC GTAGACTTGATC
ZincDz2T	AAGTACCTC TTAGTTTATGGTTGGT GTAGACTTGATC
ZincDz2A	AAGTACCTC TTAGTTTATGGTTGGA GTAGACTTGATC
ZincDz2C-v2	AAGTACCTC TCAGTTTACGGTTGGC GTAGACTTGATC
ZincDz2T-v2	AAGTACCTC TCAGTTTACGGTTGGT GTAGACTTGATC
ZincDz2A-v2	AAGTACCTC TCAGTTTACGGTTGGA GTAGACTTGATC
ZincDz2G-v2	AAGTACCTC TCAGTTTACGGTTGGG GTAGACTTGATC

**Substrates used in this study (5´→3´)**	
sub-CCAb	AGACGGATCAAGTCTAC rCrCrA GAGGTACTT-biotin
sub-CCA-mass	AGACGGATCAAGTCTAC rCrCrA GAGGTACTT
sub-CCA	AGACGGATCAAGTCTAC rCrCrA GAGGTACTT-FAM
sub-ACA	AGACGGATCAAGTCTAC rArCrA GAGGTACTT-FAM
sub-CCG	AGACGGATCAAGTCTAC rCrCrG GAGGTACTT-FAM
sub-CGA	AGACGGATCAAGTCTAC rCrGrA GAGGTACTT-FAM
sub-CCU	AGACGGATCAAGTCTAC rCrCrU GAGGTACTT-FAM
sub-CAC	AGACGGATCAAGTCTAC rCrArC GAGGTACTT-FAM
sub-CAA	AGACGGATCAAGTCTAC rCrArA GAGGTACTT-FAM
sub-CUA	AGACGGATCAAGTCTAC rCrUrA GAGGTACTT-FAM
sub-CCC	AGACGGATCAAGTCTAC rCrCrC GAGGTACTT-FAM
sub-UAC	AGACGGATCAAGTCTAC rUrArC GAGGTACTT-FAM
sub-AAG	GATCAAGTCTAC rArArG GAGGTACTT-FAM
sub-GGA	AGACGGATCAAGTCTAC rGrGrA GAGGTACTT-FAM
sub-GAA	AGACGGATCAAGTCTAC rGrArA GAGGTACTT-FAM
sub-GCA	AGACGGATCAAGTCTAC rGrCrA GAGGTACTT-FAM
sub-GUA	AGACGGATCAAGTCTAC rGrUrA GAGGTACTT-FAM
sub-AGA	AGACGGATCAAGTCTAC rArGrA GAGGTACTT-FAM
sub-AUA	AGACGGATCAAGTCTAC rArUrA GAGGTACTT-FAM
sub-UGA	AGACGGATCAAGTCTAC rUrGrA GAGGTACTT-FAM
sub-UAA	AGACGGATCAAGTCTAC rUrArA GAGGTACTT-FAM
sub-UCA	AGACGGATCAAGTCTAC rUrCrA GAGGTACTT-FAM
sub-UUA	AGACGGATCAAGTCTAC rUrUrA GAGGTACTT-FAM
sub-rCCA	AGACGGATCAAGTCTAC rCCA GAGGTACTT-FAM
miR155	rUrUrArArUrGrCrUrArArUrCrGrUrGrArUrArGrGrGrGrUrU-FAM

Supplementary References

1 Conaty, J., Hendry, P. & Lockett, T. Selected classes of minimised hammerhead ribozyme have very high cleavage rates at low Mg2+ concentration. *Nucleic Acids Res*. **27**, 2400-2407 (1999). doi:10.1093/nar/27.11.2400.

2 Fukuda, M., Kurihara, K., Tanaka, Y. & Deshimaru, M. A strategy for developing a hammerhead ribozyme for selective RNA cleavage depending on substitutional RNA editing. *RNA*. **18**, 1735-1744 (2012). doi:10.1261/rna.033399.112.

3 Santoro, S. W. & Joyce, G. F. A general purpose RNA-cleaving DNA enzyme. *Proc. Nat. Acad. Sci. U. S. A*. **94**, 4262-4266 (1997). doi:10.1073/pnas.94.9.4262.

4 Schlosser, K., Gu, J., Sule, L. & Li, Y. Sequence-function relationships provide new insight into the cleavage site selectivity of the 8-17 RNA-cleaving deoxyribozyme. *Nucleic Acids Res*. **36**, 1472-1481 (2008). doi:10.1093/nar/gkm1175.

5 Mazumdar, D. *et al.* Activity, folding and Z-DNA formation of the 8-17 DNAzyme in the presence of monovalent ions. *J. Am. Chem. Soc*. **131**, 5506-5515 (2009). doi:10.1021/ja8082939.

6 Schlosser, K., Gu, J., Lam, J. C. F. & Li, Y. In vitro selection of small RNA-cleaving deoxyribozymes that cleave pyrimidine–pyrimidine junctions. *Nucleic Acids Res*. **36**, 4768-4777 (2008). doi:10.1093/nar/gkn396.

7 He, Q. C. *et al.* Comparison of metal-ion-dependent cleavages of RNA by a DNA enzyme and a hammerhead ribozyme. *Biomacromolecules* **3**, 69-83 (2002). doi:10.1021/bm010095c.

8 Breaker, R. R. & Joyce, G. F. A DNA enzyme with Mg(2+)-dependent RNA phosphoesterase activity. *Chem. Biol*. **2**, 655-660 (1995). doi:10.1016/1074-5521(95)90028-4.

9 Ren, W. *et al.* Sensitivity of a classic DNAzyme for Pb(2+) modulated by cations, anions and buffers. *Analyst*. **145**, 1384-1388 (2020). doi:10.1039/c9an02612f.

10 Brown, A. K., Li, J., Pavot, C. M. & Lu, Y. A lead-dependent DNAzyme with a two-step mechanism. *Biochemistry*. **42**, 7152-7161 (2003). doi:10.1021/bi027332w.

11 Zhou, W., Zhang, Y., Ding, J. & Liu, J. In Vitro Selection in Serum: RNA-Cleaving DNAzymes for Measuring Ca2+ and Mg2+. *ACS Sens*. **1**, 600-606, (2016). doi:10.1021/acssensors.5b00306.

12 Feldman, A. R. & Sen, D. A new and efficient DNA enzyme for the sequence-specific cleavage of RNA. *J. Mol. Biol*. **313**, 283-294 (2001). doi:10.1006/jmbi.2001.5058.

13 Saran, R., Chen, Q. & Liu, J. Searching for a DNAzyme Version of the Leadzyme. *J. Mol. Evol*. **81**, 235-244 (2015). doi:10.1007/s00239-015-9702-z.

14 Lam, J. C., Withers, J. B. & Li, Y. A complex RNA-cleaving DNAzyme that can efficiently cleave a pyrimidine-pyrimidine junction. *J. Mol. Biol.* **400**, 689-701 (2010). doi:10.1016/j.jmb.2010.05.047.

15 Huang, P. J., Lin, J., Cao, J., Vazin, M. & Liu, J. Ultrasensitive DNAzyme beacon for lanthanides and metal speciation. *Anal. Chem*. **86**, 1816-1821 (2014). doi:10.1021/ac403762s.

16 Huang, P. J., Vazin, M., Matuszek, Z. & Liu, J. A new heavy lanthanide-dependent DNAzyme displaying strong metal cooperativity and unrescuable phosphorothioate effect. *Nucleic Acids Res*. **43**, 461-469 (2015). doi:10.1093/nar/gku1296.

17 Torabi, S. F. *et al.* In vitro selection of a sodium-specific DNAzyme and its application in intracellular sensing. *Proc. Natl. Acad. Sci. U.S.A.* **112**, 5903-5908 (2015). doi:10.1073/pnas.1420361112.

18 Zhou, W., Saran, R., Chen, Q., Ding, J. & Liu, J. A New Na(+)-Dependent RNA-Cleaving DNAzyme with over 1000-fold Rate Acceleration by Ethanol. *ChemBioChem*. **17**, 159-163, (2016). doi:10.1002/cbic.201500603.

19 Saran, R. & Liu, J. A Silver DNAzyme. *Anal. Chem*. **88**, 4014-4020 (2016). doi:10.1021/acs.analchem.6b00327.
