# Supplementary material for: Zn2+-dependent DNAzymes that cleave all combinations of ribonucleotides
Source: Commun Biol. 2021 Feb 16;4:221. doi: 10.1038/s42003-021-01738-6 (PMC7886857; doi:10.1038/s42003-021-01738-6)
Supplement: Supplementary file 3 — Description of Additional Supplementary Files [file 42003_2021_1738_MOESM3_ESM.pdf]

Description of additional supplementary items

File Name: Supplementary Data 1

Description: Source data for the main and supplementary figures

Source Data Fig. 1

(c) Sequences that constitutes Motif 1 and Motif 2 in MEME analysis.

(e) The full gel image in figure 1e.

Source Data Fig. 2

(a-d) Gel images and percentage values of cleaved substrates calculated by eq 1.

Source Data Fig. 3

(a) Gel images and percentage values of cleaved substrates calculated by eq 1.

Source Data Fig. 4

(a, b) Gel images and percentage values of cleaved substrates calculated by eq 1.

Source Data Fig. 5

(b) Gel images and percentage values of cleaved substrates calculated by eq 1.

Source Data Table 1

Gel images and percentage values of cleaved substrates calculated by eq 1.

Source Data Supplementary Fig. 2

Gel images and percentage values of cleaved substrates calculated by eq 1.

Source Data Supplementary Fig. 3

Gel images and percentage values of cleaved substrates calculated by eq 1.

Source Data Supplementary Fig. 4

Gel images and percentage values of cleaved substrates calculated by eq 1.

Source Data Supplementary Fig. 5

Gel images and percentage values of cleaved substrates calculated by eq 1.

Source Data Supplementary Fig. 6

Gel images and percentage values of cleaved substrates calculated by eq 1.
